# Endothelial-like cells in chronic thromboembolic pulmonary hypertension: crosstalk with myofibroblast-like cells

**DOI:** 10.1186/1465-9921-12-109

**Published:** 2011-08-22

**Authors:** Seiichiro Sakao, Hiroyuki Hao, Nobuhiro Tanabe, Yasunori Kasahara, Katsushi Kurosu, Koichiro Tatsumi

**Affiliations:** 1 Department of Respirology (B2), Graduate School of Medicine, Chiba University, 1-8-1 Inohana, Chuo-ku, Chiba 260-8670, Japan; 2 Department of Surgical Pathology, Hyogo College of Medicine, 1-1 Mukogawa-cho, Nishinomiya, Hyogo, 663-8501, Japan

**Keywords:** neointima, myofibroblast, endothelial cells, CTEPH.

## Abstract

**Background:**

Chronic thromboembolic pulmonary hypertension (CTEPH) is characterized by intravascular thrombus formation in the pulmonary arteries.

Recently, it has been shown that a myofibroblast cell phenotype was predominant within endarterectomized tissues from CTEPH patients. Indeed, our recent study demonstrated the existence of not only myofibroblast-like cells (MFLCs), but also endothelial-like cells (ELCs). Under *in vitro *conditions, a few transitional cells (co-expressing both endothelial- and SM-cell markers) were observed in the ELC population. We hypothesized that MFLCs in the microenvironment created by the unresolved clot may promote the endothelial-mesenchymal transition and/or induce endothelial cell (EC) dysfunction.

**Methods:**

We isolated cells from these tissues and identified them as MFLCs and ELCs. In order to test whether the MFLCs provide the microenvironment which causes EC alterations, ECs were incubated in serum-free medium conditioned by MFLCs, or were grown in co-culture with the MFLCs.

**Results:**

Our experiments demonstrated that MFLCs promoted the commercially available ECs to transit to other mesenchymal phenotypes and/or induced EC dysfunction through inactivation of autophagy, disruption of the mitochondrial reticulum, alteration of the SOD-2 localization, and decreased ROS production. Indeed, ELCs included a few transitional cells, lost the ability to form autophagosomes, and had defective mitochondrial structure/function. Moreover, rapamycin reversed the phenotypic alterations and the gene expression changes in ECs co-cultured with MFLCs, thus suggesting that this agent had beneficial therapeutic effects on ECs in CTEPH tissues.

**Conclusions:**

It is possible that the microenvironment created by the stabilized clot stimulates MFLCs to induce EC alterations.

## Background

It is generally known that chronic thromboembolic pulmonary hypertension (CTEPH) is one of the leading causes of severe pulmonary hypertension. CTEPH is characterized by intravascular thrombus formation and fibrous stenosis or complete obliteration of the pulmonary arteries [1]. The consequence is increased pulmonary vascular resistance, resulting in pulmonary hypertension and progressive right heart failure. Pulmonary endarterectomy (PEA) is the current mainstream of therapy for CTEPH [2]. Moreover, recent studies have provided evidence suggesting that, although CTEPH is believed to result from acute pulmonary embolism [3,4], small-vessel disease appears and worsens later in the course of disease [5]. Histopathologic studies of microvascular changes in CTEPH have shown indistinguishable vascular lesions from those seen in idiopathic pulmonary arterial hypertension (IPAH) and Eisenmenger's syndrome [6-8]. Especially *in vitro *and *ex vivo *experiments, pulmonary artery endothelial cell (EC) in the group of pulmonary hypertensive diseases are suggested to exhibit an unusual hyperproliferative potential with decreased susceptibility to apoptosis [9,10], indicating that dysfunctional EC may contribute to the progression of the diseases.

Recently, Firth et al showed that multipotent mesenchymal progenitor cells are present in endarterectomized tissues from patients with CTEPH, and that a myofibroblast cell phenotype was predominant within these tissues, contributing extensively to the vascular lesion/clot [11]. Indeed, we have also demonstrated the existence of not only myofibroblast-like cells (MFLCs), but also endothelial-like cells (ELCs) in these tissues [12]. Under *in vitro *conditions, morphological alterations were more easily detected in the ELCs. Smooth muscle (SM)-like cells (defined by their expression of α-SM-actin (SMA)) and a few transitional cells (co-expressing both endothelial- (von Willebrand factor) and SM- (α-SMA) cell markers) were consistently observed by immunohistochemical staining (preliminary data).

*In vitro *experiments conducted to assess the contribution of ECs to the development of pulmonary arterial hypertension (PAH) have demonstrated that the shift to a transdifferentiated phenotype could be attributed to selection of distinct cell subpopulations (i.e., stem-like cells). These findings also suggest that the endothelial-mesenchymal transition (EnMT) might be an important contributor to pathophysiological vascular remodeling in the complex vascular lesions of PAH [13], because, although bone marrow-derived cells could participate in arterial neointimal formation after mechanical injury, they did not contribute substantially to pulmonary arterial remodeling in an experimental PAH model [14].

Autophagy is a catabolic process involving the degradation of intracellular material that is evolutionarily conserved between all eukaryotes. During autophagy, cytoplasmic components are engulfed by double-membrane-bound structures (autophagosomes) and delivered to lysosomes/vacuoles for degradation [15]. Recent studies indicate that autophagy plays an important role in many different pathological conditions. Indeed, both activation and inactivation of autophagy may impact cancer cell growth. If autophagy cannot be activated, protein synthesis predominates over protein degradation, and tumor growth is stimulated. In contrast, autophagy may be activated in more advanced stages of cancer to guarantee the survival of cells in minimally-vascularized tumors [16].

The interactions between ECs and smooth muscle cells (SMCs), which exist in close contact via a functional syncytium, are involved in the process of new vessel formation that occurs during development, as part of wound repair, and during the reproductive cycle [17-19]. We hypothesized that MFLCs stimulated by the microenvironment created by the unresolved clot may promote ECs to transit to other mesenchymal phenotypes and/or induce EC dysfunction, contributing to the vascular lesion, i.e., not only proximal vasculature, but also microvascular. In the experiments considered here, we isolated cells from endarterectomized tissue from patients with CTEPH and identified them as MFLCs and ELCs. In order to show the hypothesis, human pulmonary microvascular ECs were incubated in a serum-free medium conditioned by MFLCs, or ECs were co-cultured with MFLCs. The aim of this study was to examine whether MFLCs in the microenvironment created by the unresolved clot can, in principle, affect EC disorder through the EnMT and autophagy.

## Methods

### Cell lines and reagents

The PEA tissues of patients with CTEPH were obtained following PEA performed by Dr. Masahisa Masuda at the Chiba Medical Center, Japan. Control pulmonary arteries were obtained following lung resection for peripheral cancer by Dr. Ichiro Yoshino at the Chiba University Hospital, Japan. Written informed consent was acquired before surgery from all patients from whom tissue samples were obtained. The study was approved by the Research Ethics Committee of Chiba University School of Medicine, and all subjects gave their informed consent in writing. Although not clinically accurate, the PEA tissues were defined as mentioned below. PEA samples obtained from the region directly surrounding the fibrotic clot are referred to as "proximal" vascular tissue and those obtained from areas after the fibrotic clot region are referred to as the "distal" vascular tissue [11]. The tissues were cultured and various explant outgrowth cells were dissociated as described previously [12]. Myofibroblast-like cells (MFLCs) and endothelial-like cells (ELCs) were isolated and identified from endarterectomized tissue from patients with CTEPH and pulmonary arterial fibroblast-like cells from control pulmonary arteries. PEA samples obtained from a total of six patients undergoing PEA were examined in this study.

Human pulmonary microvascular ECs were obtained from Lonza Inc (Allendale, NJ, USA). The following antibodies were used during our present studies: mouse anti-α-SMA (1:1000, Sigma, St. Louis, MO, USA), mouse anti-vimentin (1:200, DAKO, Carpinteria, CA, USA), mouse anti-human desmin (1:100, DAKO, Carpinteria, CA, USA), anti-mouse IgG Ab conjugated with Rhodamine dye (1:500, Molecular Probes, Eugene, OR, USA), rabbit anti-von Willebrand factor (Factor VIII) (1:1000, DAKO, Carpinteria, CA, USA), anti-rabbit IgG conjugated with Alexa-488 fluorescent dye (1:500, Molecular Probes, Eugene, OR, USA), and rabbit anti-CD31 (1:1000, DAKO, Carpinteria, CA, USA). Rapamycin was purchased from Merck (Frankfurter, Germany).

### Immunofluorescence staining

The cells were fixed in a 1:1 mixture of methanol and acetone for 2 minutes followed by blocking with normal goat serum for 30 minutes as described previously [13]. The cells were incubated with primary antibodies (anti-α-smooth muscle actin (SMA), anti-von Willebrand factor, anti-vimentin and anti-desmin) for 1 hour at room temperature, and then with secondary antibodies (anti-mouse IgG conjugated with Alexa-594 fluorescent dye and anti-rabbit IgG conjugated with Alexa-488 fluorescent dye) for 1 hour at room temperature. Stained cells were embedded in VectaShield mounting medium with DAPI (Vector Laboratories, Burlingame, CA, USA) and were examined with a NIKON Eclipse 80 i microscope (Nikon, Tokyo, Japan) using the VB-7210 imaging system (Keyence, Tokyo, Japan). Positive cells were counted in 3 different fields at a magnification of × 200 using a fluorescence microscope.

### Double immunohistochemical staining

Endarterectomized samples were embedded in optimal cutting temperature (OCT) compound (Sakura Tissue Tek), frozen, and cut into 10- μm sections with a cryostat. For basic characterization, standard hematoxylin and eosin (H & E) staining was performed. The CD31 antibody was used to stain endarterectomized tissue, together with αSMA to stain transitional cells. αSMA staining (blue) was developed with alkaline phosphatase-conjugated secondary antibody, and then CD31 staining (brown) was developed with peroxidase-conjugated secondary antibody. Transitional cells were confirmed by αSMA positively stained cytosol that also had concomitant positive cytoplasmic staining in CD31 positive cells.

### ELISA (Enzyme-Linked ImmunoSorbent Assay)

TGF-β_1 _were measured by sandwich ELISA techniques by ELISA Tech (Aurora, CO, USA) utilizing reagents from R&D systems (Minneapolis, MN, USA). The samples were read in a spectrophotometer at 405 nm. Antibodies and tracer were bought from Cayman Chemicals (Ann Arbor, Mi, USA).

### Human pulmonary microvascular ECs in the conditioned medium

At passage 2 MFLCs or pulmonary arterial fibroblast-like cells were seeded at a density of 1.5 × 10^4 ^cells/cm^2 ^and were subcultured when they were to 90% confluences (4-8 days). They were washed 3 times using phosphate-buffered saline (PBS) and were incubated with serum-free medium for 48 hours. HPMVEC were seeded in 6 cm dishes at 1 × 10^5 ^density and cultured in EGM supplemented with 5% fetal bovine serum. At 70 to 80% confluence they were washed 3 times with PBS, incubated in the conditioned medium for 48 hours and incubated in EGM again for 48 hours. After the incubation periods, they were assessed microscopically, further characterized by immunohistochemical staining and harvested to extract RNA for quantitative RT-PCR and to extract protein for ELISA.

### Co-culture of human pulmonary microvascular ECs and MFLCs

Co-culture of human pulmonary microvascular ECs and MFLCs was done on a 6-well plate (BD Falcon) with Cell Culture Inserts (Falcon, 353102, 1.0 microns pore size). Human pulmonary microvascular ECs or pulmonary arterial fibroblast-like cells (at 5 × 10^4 ^density) and MFLCs (at 5 × 10^4 ^density) were added into the lower or upper chamber with or without rapamycin (10 nM). After two weeks incubation periods, they were assessed microscopically and further characterized by immunohistochemical staining, harvested to extract RNA for PCR array, and other assays.

### Magnetic cell sorting (MACS)

After trypsinization of ECLCs at passage 2, CD31 positive cells were isolated by using CD31 MicroBeads (Direct CD31 progenitor cell isolation kit, Miltenyi Biotec Inc, Auburn, CA, USA) as described previously [13]. After trypsinization of ECLCs at passage 2, 100 μl of FcR Blocking Reagent (Direct CD31 progenitor cell isolation kit, Miltenyi Biotec Inc, Auburn, CA, USA) per 10^8 ^total cells was added to the cell suspension to inhibit nonspecific or Fc-receptor mediated binding of CD31 MicroBeads (Direct CD31 progenitor cell isolation kit, Miltenyi Biotec) to non-target cells. Cells were labeled by adding 100 μl CD31 MicroBeads per 10^8 ^total cells, and incubated for 30 min at 6-12°C. After washing, cells were resuspended in 500 μl buffer and applied to the MS+/RS+ column with the column adapter in the magnetic field of the MACS separator. The column was washed 3× with 500 μl buffer. The column was removed from the separator and the retained cells were flushed out with 1 ml buffer under pressure using the plunger supplied with the column. The cells were incubated in EGM and cultured until passage 5.

### Total RNA isolation and Quantitative measurement

Total RNA was extracted from human pulmonary microvascular ECs with an RNeasy Mini Kit (Qiagen, CA, USA). RNA and cRNA yields were quantitated on a Nano-Drop ND-1000 UV-Vis Spectrophotometer (NanoDrop Technologies, Wilmington, DE, USA) as described previously [13].

### PCR array analysis

RT^2 ^Profiler™ PCR Arrays (SABiosciences, Frederick, USA) are the reliable and sensitive tools for analyzing the expression of a focused panel of genes in signal transduction pathways, biological process or disease related gene networks. The 96-well plate Human Autophagy PCR-array (PAHS-084) which profiles the expression of 84 key genes involved in autophagy and Human Endothelial Cell Biology PCR-array (PAHS-015) which profiles the expression of 84 genes related to endothelial cell biology were selected as the hypothesis.

There is a better sensitivity of quantitative PCR in comparison to microarray [20,21]. The PCR Arrays can be used for research on various disease including cancer, immunology, and phenotypic analysis of cells.

The mRNA of each co-cultured EC was converted into cDNA using the RT^2 ^First Strand Kit (SABiosciences, Frederick, USA). This cDNA was then added to the RT^2 ^SYBR Green qPCR Master Mix (SABiosciences, Frederick, USA). Next, each sample was aliquotted on PCR-arrays. All steps were done according to the manufacturer's protocol for the ABI Prism 7000 Sequence Detection System. To analyze the PCR-array data, an MS-Excel sheet with macros was downloaded from the manufacturer's website http://www.sabiosciences.com/pcrarraydataanalysis.php. The website also allowed online analysis. For each PCR reaction, the excel sheet calculated two normalized average C_t _values, a paired *t *test *P *value and a fold change. Data normalization was based on correcting all C_t _values for the average C_t _values of several constantly expressed housekeeping genes (HKGs) present on the array. PCR-array analysis results were evaluated.

### SMAD reporter assay

The SMAD reporter assay detects the activity of TGFβ signaling pathway through monitoring the SMAD transcriptional response in cultured cells. Cignal SMAD Reporter (GFP) Kit (SABiosciences, Frederick, USA) was adapted to assess the activity of this signaling pathway.

Co-cultured human pulmonary microvascular ECs with pulmonary arterial fibroblast-like cells or MFLCs were trypsinized, suspended at 1 × 10^4^/well at density, and seeded into 96-well cell culture plates. Transfection complexes including the signal reporters were aliquoted into wells containing overnight cell cultures. After 40 hours of transfection, expression of the GFP reporter was monitored via the fluorometry (Infinite 200 PRO, Tecan Group Ltd., Männedorf, Switzerland). All steps were done according to the manufacturer's protocol.

#### Reactive oxygen species (ROS) assay

Measuring ROS activity intracellularly, we adapted OxiSelect ROS assay kit (Cell Biolabs, Inc., San Diego, USA).

Co-cultured human pulmonary microvascular ECs with pulmonary arterial fibroblast-like cells or MFLCs were trypsinized, suspended at 1 × 10^4^/well at density, and seeded into 96-well cell culture plates. Media was removed from all wells and cells were washed with DPBS 3 times. 100 μL of 1 × 2,7-dichlorofluorescein diacetate (DCFH)-DA/media solution added to cells and they were incubated at 37° for 60 minutes. Solution was removed and cells were washed with DPBS 3 times. DCFH-DA loaded cells were treated with hydrogen peroxide (100 μM) in 100 μL medium. After 1 hour, the fluorescence was read via the fluorometry (Infinite 200 PRO, Tecan Group Ltd., Männedorf, Switzerland). All steps were done according to the manufacturer's protocol.

### Statistical analysis

Three independent experiments were performed and subjected to statistical analysis. The results were expressed as the means ± SEM. PCR array data were analyzed using a paired *t *test according to the manufacturer's protocol and other data were the Mann-Whitney U test. A p < 0.05 was considered to be significant for all comparisons.

## Results

### The cellular composition of endarterectomized tissue from CTEPH patients

**Figure 1:**
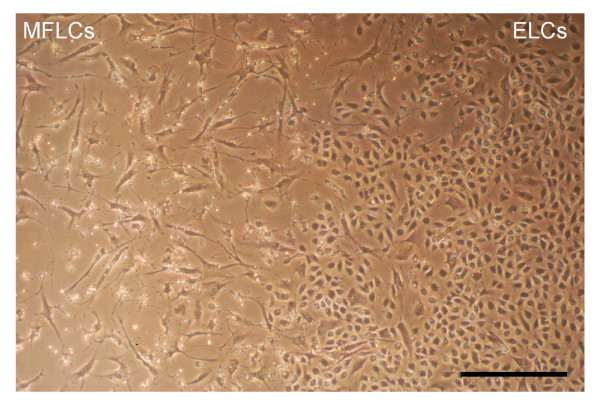
**Cells from endarterectomized tissue**. The MFCsL and ELCs from endarterectomized tissue were microscopically assessed. The magnification was 100×. Scale bar = 100 μm; MFLCs = myofibroblast-like cells; ELCs = endothelial-like cells.

Two different cell types were isolated from the "distal" vascular tissue in the patients with CTEPH. The cell types were determined by morphology to be ELCs (rounded appearance and cell-cell contact in the monolayer) and MFLCs (spindle-shaped with cytoplasmic extensions) (Figure 1). They were dissociated and passaged free from surrounding cells using cloning cylinders. MFLCs were prepared from each of the six patients and ELCs could be isolated from 4 of the six patients.

Moreover, another cell type was isolated from the vessel wall tissues of control pulmonary arteries, defined morphologically as fibroblast-like cells (pulmonary arterial fibroblast-like cells) (data not shown). These cells were used as control cells, and were prepared in the same way as the CTEPH specimens.

The cells outgrown from the organized thrombotic tissue and control pulmonary arteries were further characterized by immunohistochemical staining for desmin, vimentin, von Willebrand factor (Factor VIII) andα-SMA. ELCs were positively stained for the endothelial cell (EC)-specific marker (Factor VIII) and the mesenchymal-specific marker (vimentin) and negative for the 2 smooth muscle cell (SMC)-specific markers (desmin and α-SMA) [12]. MFLCs were Factor VIII and desmin negative and vimentin and α-SMA positive [12]. Pulmonary arterial fibroblast-like cells were Factor VIII, desmin and α-SMA negative, and vimentin positive (data not shown).

### Phenotypic alteration of ELCs

**Figure 2:**
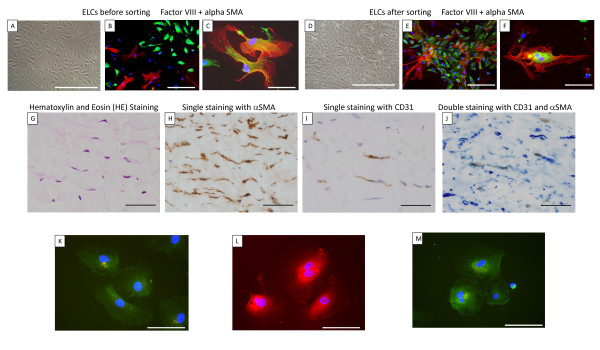
**ELCs from endarterectomized tissue**. A-F), ELCs were assessed by immunofluorescence staining for anti-Factor VIII (green) and anti-α-SMA (red) to confirm the phenotypes of the cells. A), B) and C), ELCs before sorting; D), E) and F), ELCs after sorting; A) and D), the magnification was 100×. Scale bar = 100 μm; B) and E), the magnification was 200×. Scale bar = 50 μm; C) and F), the magnification was 400×. Scale bar = 25 μm. The blue staining was DAPI. G-J), Immunohistochemical staining of endarterectomized tissue. The neointimal layer of distal vascular wall tissues was assessed by immunohistochemical staining. G), Hematoxylin and Eosin (HE) staining; H), Single staining for α-SMA; I), Single staining for CD31; J), Double staining for CD31 and α-SMA; the magnification was 200×. Scale bar = 50 μm. K, L, M), Immunofluorescence staining of ELCs for the autophagic marker, LC3 (K), mitochondrial marker mitotracker red (L), and SOD-2 (M). K), The formation of autophagosomes (green punctate structures) was not detected. L), The normal filamentous mitochondrial reticulum (red punctate structures) was not detected. M), SOD-2 expression (green punctate structures) was not detected. The blue staining was DAPI. The magnification was 400×. Scale bar = 25 μm. ELCs = endothelial-like cells.

After a few passages, morphological alterations were detected in the ELCs. The cell-cell contact of the endothelial monolayers became disrupted, and some ELCs had lost their rounded appearance and acquired an elongated, mesenchymal-like morphology. At the 2nd passages, the morphological alterations could not to be detected microscopically (Figure 2A), but some SM-like cells (as defined by expression of α-SMA) (Figure 2B) and a few transitional cells (co-expressing both endothelial- and SM-cell markers) were consistently observed (Figure 2C) by immunohistochemical staining. These transitional cells could be observed in ELCs prepared from 4 of the six samples.

Since this result suggested that ELCs were contaminated with SMCs, at the 3rd passage, they were sorted for the EC marker CD31 in order to establish that the ELCs were free of contamination with SMCs. After magnetic cell sorting for the EC marker CD31, ELCs were examined microscopically, and unusual "pile" growth and disrupted formation of the endothelial monolayer were detected (Figure 2D). Moreover, SM-like cells (Figure 2E) and transitional cells were consistently observed (Figure 2F).

### Transitional cells in endarterectomized CTEPH tissue

To detect transitional cells which co-express both endothelial (CD31) and SM (α-SMA) markers in the PEA tissues of patients with CTEPH, a double immunostaining method for CD31/α-SMA was performed. The HE staining of the neointimal layers of both the "proximal" and the "distal" vascular tissues indicated the presence of a fibrin network, and nuclei are seen within this region (Figure 2G). These neointimal layers are composed of some α-SMA positive cells (Figure 2H). Although the neointimal layers of both the "proximal" and the "distal" vascular tissues were composed of α-SMA positive cells, CD31 positive cells were found in the "distal" vascular wall tissue but not in the "proximal" vascular tissue (Figure 2I). As shown in Figure 2J, a few CD31 and α-SMA double-positive cells were identified in the "distal" vascular tissues, thus indicating the presence of "intermediate" cells, which were intermediate between ECs and muscle cells in structure, in the neointimal lesions of CTEPH patients.

### Decreased expression of Autophagic marker LC3 (microtubule-associated protein1 light chain 3; MAP1LC3), abnormal mitochondria, and decreased expression of superoxide dismutase (SOD)-2 in ELCs

To assess ELC alterations, an immunofluorescence staining method for LC3, mitochondrial marker mitotracker red, and SOD-2 was performed.

LC3 is a major constituent of the autophagosome, a double-membrane structure that sequesters the target organelle/protein and then fuses with endo/lysosomes where the contents and LC3 are degraded. Confocal microscopy showed that the ELCs did not express LC3. The formation of autophagosomes (green punctate structures) was not detected in these cells (Figure 2K).

SOD-2 is an enzyme that catalyzes the dissociation of superoxide into oxygen and hydrogen peroxide. As such, this is an important antioxidant defense in nearly all cells exposed to oxygen and is located in the mitochondria. Immunofluorescence staining for mitochondrial marker mitotracker red revealed that the normal filamentous mitochondrial reticulum was disrupted and rarefied in ELCs (Figure 2L). Moreover, SOD-2 was decreased in ELCs (Figure 2M).

### Phenotypic alteration of human pulmonary microvascular ECs is induced by MFLCs-conditioned medium

As mentioned above, ELCs isolated from the PEA tissues could easily change their phenotype during passaging. We postulated that the interactions of ELCs and MFLCs, which exist in close contact in the PEA tissues, are involved in a process of organized thrombus formation that occurs during the development of CTEPH. One basic component of this interaction may be the MFLC-induced transition of ELCs. To test this hypothesis, the commercially available human pulmonary microvascular ECs were incubated in serum-free medium conditioned by MFLCs to determine whether MFLCs release mediators which cause phenotypic alteration of human pulmonary microvascular ECs.

**Figure 3:**
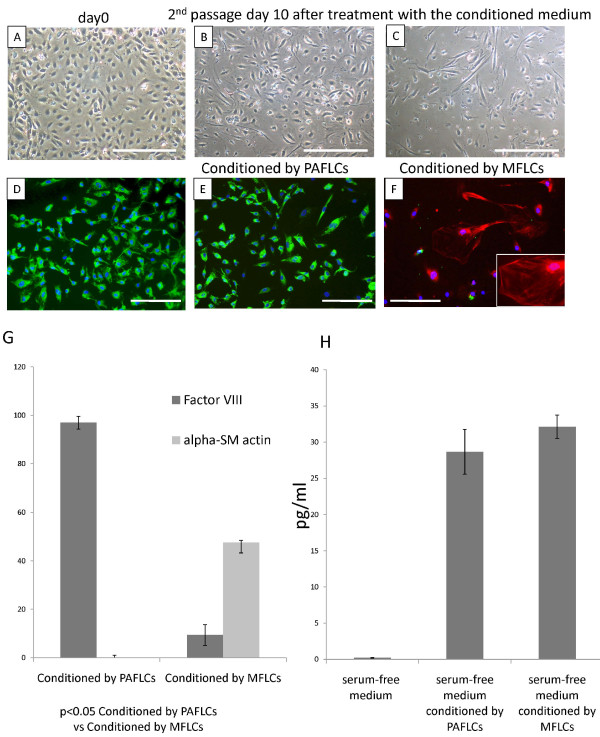
**Human pulmonary microvascular ECs (HPMVECs) in serum-free medium conditioned by pulmonary arterial fibroblast-like cells (PAFLCs) or myofibroblast-like cells (MFLCs)**. The phenotypic alteration of HPMVECs was assessed microscopically and by immunofluorescence staining. A) and D), Before incubation in serum-free medium conditioned by PAFLCs and MFLCs; B) and E), At the 2nd passage after incubation in serum-free medium conditioned by PAFLCs; C) and F), At the 2nd passage after incubation in serum-free medium conditioned by MFLCs; A), B) and C), microscopic findings; the magnification was 100×. Scale bar = 100 μm; D), E) and F), Immunofluorescence staining for anti-Factor VIII (green) and anti-α-SMA (red). The blue staining was DAPI. The magnification was 200×. Scale bar = 50 μm. F), Some cells were positive for smooth muscle actin fibers (see inset); HPMVECs = human pulmonary microvascular endothelial cells; MFLCs = myofibroblast like cells; PAFLCs = fibroblast-like cells from control pulmonary arteries. G) Positive cells for anti-von Willebrand factor and anti-α-SM-actin were counted in 3 different fields at a magnification of × 200 in a fluorescence microscope. *P < 0.05 _VS. _PAFLCs, n ≥ 3. H) The TGF-β1 protein levels in the conditioned medium were measured by ELISA. There were no significant differences between the serum-free medium conditioned by PAFLCs and MFLCs.

We first established that the human pulmonary microvascular ECs were free of contamination with vascular smooth muscle cells (VSMCs) by morphology (rounded appearance and cell-cell contact of the monolayer) (Figure 3A) and by immunofluorescence staining using anti-von Willebrand factor (Figure 3D), anti-α-SMA (Figure 3D), anti-vimentin (data not shown), and anti-human desmin (data not shown) antibodies. The endothelial cell-specific marker and the mesenchymal-specific marker were positive, and the 2 smooth muscle-specific markers were negative, providing evidence that the human pulmonary microvascular ECs were not contaminated with VSMCs.

At the 2nd passage after incubation in serum-free medium conditioned by pulmonary arterial fibroblast-like cells and MFLCs, the phenotypic alteration of human pulmonary microvascular ECs was assessed microscopically and by immunofluorescence staining. The cell-cell contact of the endothelial monolayers became disrupted, and many ECs had lost their rounded appearance and acquired an elongated, mesenchymal-like morphology in the medium conditioned by MFLCs (Figure 3C) in comparison to the medium conditioned by pulmonary arterial fibroblast-like cells (Figure 3B). The number of ECs (as defined by expression of von Willebrand factor) decreased, and SM-like cells (as defined by expression of α-SMA) were consistently observed in the medium conditioned by MFLCs (Figure 3F, G), but not in the medium conditioned by pulmonary arterial fibroblast-like cells (Figure 3E, G).

### Expression of TGF-β1 protein in the conditioned medium

Because TGF-β1 is known to be involved in inducing the endothelial-mesenchymal transition [22] and is known to promote α-SMA expression in non-muscle cells (ECs and fibroblasts derived from various tissues) [23,24], the protein levels in the conditioned medium were measured by ELISA. Serum-free medium conditioned by MFLCs contained higher TGF-β1 levels than medium conditioned by pulmonary arterial fibroblast-like cells, but the difference was not statistically significant (Figure 3H).

### Phenotypic alteration of human pulmonary microvascular ECs co-cultured with MFLCs

**Figure 4:**
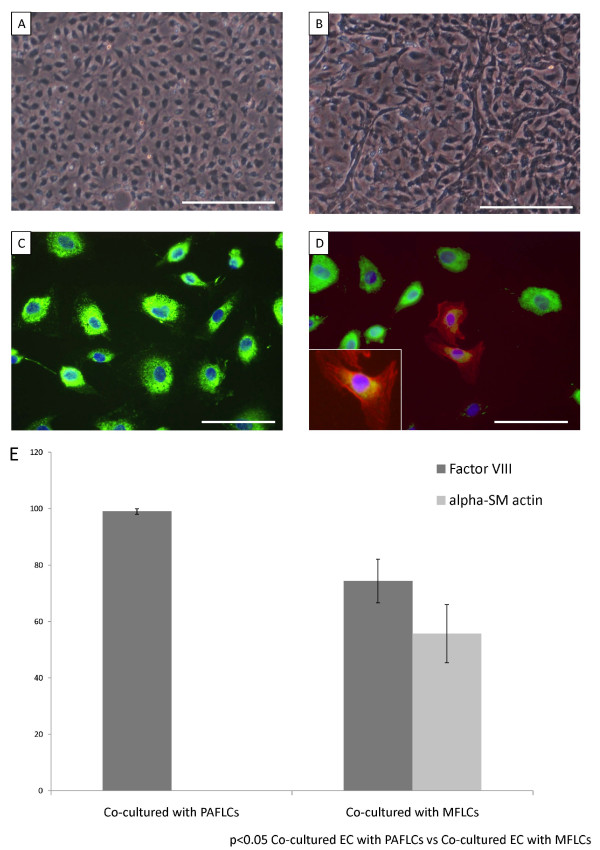
**Human pulmonary microvascular ECs (HPMVECs) co-cultured with pulmonary arterial fibroblast-like cells (PAFLCs) or myofibroblast-like cells (MFLCs)**. The phenotypical alteration of HPMVECs was assessed microscopically and by immunofluorescence staining after a 14 day incubation period. A) and C), HPMVECs co-cultured with PAFLCs; B) and D), HPMVECs co-cultured with MFLCs; A) and B), Microscopic findings; the magnification was 100×. Scale bar = 100 μm; C) and D), Immunofluorescence staining for anti-Factor VIII (green) and anti-α-SMA (red). The blue staining was DAPI. The magnification was 400×. Scale bar = 25 μm. D), Some cells coexpressed both anti-Factor VIII and anti-α-SMA (see inset); HPMVECs = human pulmonary microvascular endothelial cells; MFLCs = myofibroblast-like cells; PAFLCs = fibroblast-like cells from control pulmonary arteries. E) Positive cells for anti-von Willebrand factor and anti-α-SM-actin were counted in 3 different fields at a magnification of × 200 in a fluorescence microscope. *P < 0.05 _VS. _PAFLCs, n ≥ 3.

After a 14 day incubation period, morphological alterations were detected in human pulmonary microvascular ECs co-cultured with MFLCs (Figure 4B, D), but not those cultured with pulmonary arterial fibroblast-like cells (Figure 4A, C). The cell-cell contact of the endothelial monolayers (Figure 4A) became disrupted, and hill and valley formation appeared. Moreover, some ECs had lost their rounded appearance and acquired an elongated, mesenchymal-like morphology (Figure 4B). Some SM-like cells (as defined by their expression of α-SMA) and a few transitional cells (co-expressing both endothelial- and SM- cell markers) were consistently observed (Figure 4D, E) by immunohistochemical staining.

### Autophagy PCR array analysis of human pulmonary microvascular ECs co-cultured with MFLCs

**Figure 5:**
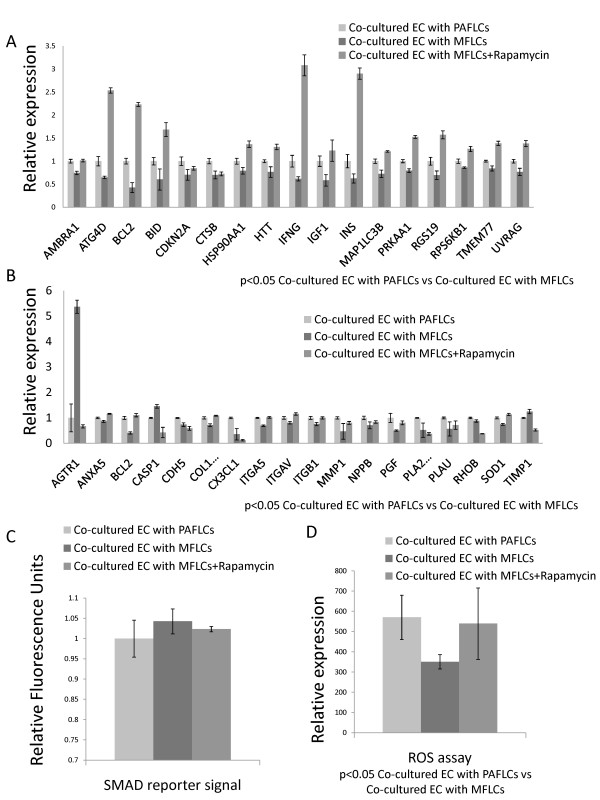
**Human pulmonary microvascular ECs (HPMVECs) co-cultured with pulmonary arterial fibroblast-like cells (PAFLCs) or myofibroblast-like cells (MFLCs)**. Autophagy and Endothelial cell biology. A) Autophagy PCR array analysis of HPMVECs co-cultured with PAFLCs, MFLCs or MFLCs+Rapamycin. There were decreases in the expression of 17 autophagy-related genes in the ECs co-cultured with MFLCs in comparison those co-cultured with PAFLCs (P < 0.05; *n *= 3). This result is related to 3 different patients out of six of co-culture or conditioned medium. See table 1 for definitions of the abbreviations. B) Endothelial cell biology PCR array analysis of HPMVECs co-cultured with PAFLCs, MFLCs or MFLCs+Rapamycin. There were decreases in 15 and increases of 3 genes in ECs co-cultured with MFLCs in comparison to the expression in ECs co-cultured with PAFLCs (P < 0.05; *n *= 3). This result is related to 3 different patients out of six of co-culture or conditioned medium. See table 2 and 3 for the definitions. C) SMAD reporter signal in HPMVECs co-cultured with MFLCs. There was no statistical difference in the expression of SMAD reporter signal in ECs co-cultured with MFLCs in comparison to the expression in those co-cultured with PAFLCs treated with or without rapamycin. D) Accumulation of ROS in HPMVECs co-cultured with MFLCs. The decreased production of ROS has been detected in ECs co-cultured with MFLCs in comparison to the expression in those co-cultured with PAFLCs (P < 0.05; *n *= 3). Although there was a tendency that rapamycin treatment of ECs co-cultured with MFLCs reversed the decreased production of ROS, there was no statistical difference between them.

There were decreases in the expression of 17 autophagy-related genes in ECs co-cultured with MFLCs in comparison to the expression in ECs co-cultured with pulmonary arterial fibroblast-like cells (Figure 5A) (Table 1). Four of these genes; AMBRA1, ATG4D, MAP1LC3B, and RGS19, are involved in autophagic vacuole formation. In particular, ATG4D is responsible for protein targeting to the membrane/vacuole, and is responsible for protein transport and protease activity. Ten of the 17 genes; BCL2, BID, CDKN2A, CTSB, HSP90AA1, HTT, IFNG, IGF1, INS, and PRKAA1 are co-regulators of autophagy and apoptosis. Three genes; RPS6KB1, TMEM77, and UVRAG are related to autophagy in response to other intracellular signals.

**Table 1:** Autophagy PCR array

Biological process description	Gene name	Gene symbol	Public ID	P-value
Autophagy Machinary Components: Genes Involved in Autophagic Vacuole Formation	Autophagy/beclin-1 regulator 1	AMBRA1	NM_017749	0.00308
Autophagy Machinary Components: Genes Involved in Autophagic Vacuole Formation Genes Responsible for Protein Targeting to Membrane/Vacuole Genes Responsible for Protein Transport Genes with Protease Activity	ATG4 autophagy related 4 homolog D (S. cerevisiae)	ATG4D	NM_032885 NM_017749	0.01167
Regulation of Autophagy: Co-Regulators of Autophagy and Apoptosis	B-cell CLL/lymphoma 2	BCL2	NM_000633	0.000727
Regulation of Autophagy: Co-Regulators of Autophagy and Apoptosis	BH3 interacting domain death agonist	BID	NM_001196	0.047933
Regulation of Autophagy: Co-Regulators of Autophagy and Apoptosis	Cyclin-dependent kinase inhibitor 2A (melanoma, p16, inhibits CDK4)	CDKN2A	NM_000077	0.044888
Regulation of Autophagy: Co-Regulators of Autophagy and Apoptosis	Cathepsin B	CTSB	NM_001908	0.010802
Regulation of Autophagy: Chaperone-Mediated Autophagy	Heat shock protein 90 kDa alpha (cytosolic), class A member 1	HSP90AA1	NM_001017963	0.037151
Regulation of Autophagy: Co-Regulators of Autophagy and Apoptosis	Huntingtin	HTT	NM_002111	0.033212
Regulation of Autophagy: Co-Regulators of Autophagy and Apoptosis Co-Regulators of Autophagy and the Cell Cycle	Interferon, gamma	IFNG	NM_000619	0.017749
Regulation of Autophagy: Co-Regulators of Autophagy and Apoptosis	Insulin-like growth factor 1 (somatomedin C)	IGF1	NM_000618	0.017282
Regulation of Autophagy: Co-Regulators of Autophagy and Apoptosis	Insulin	INS	NM_000207	0.045037
Autophagy Machinary Components: Genes Involved in Autophagic Vacuole Formation	Microtubule-associated protein 1 light chain 3 beta	MAP1LC3B	NM_022818	0.011251
Regulation of Autophagy: Co-Regulators of Autophagy and Apoptosis Autophagy in Response to Other Intracellular Signals	Protein kinase, AMP-activated, alpha 1 catalytic subunit	PRKAA1	NM_006251	0.005633
Autophagy Machinary Components: Genes Involved in Autophagic Vacuole Formation	Regulator of G-protein signaling 19	RGS19	NM_005873	0.021592
Regulation of Autophagy: Autophagy in Response to Other Intracellular Signals	Ribosomal protein S6 kinase, 70 kDa, polypeptide 1	RPS6KB1	NM_003161	0.024072
Regulation of Autophagy: Autophagy in Response to Other Intracellular Signals	Transmembrane protein 77	TMEM77	NM_178454	0.019285
Regulation of Autophagy: Autophagy in Response to Other Intracellular Signals	UV radiation resistance associated gene	UVRAG	NM_003369	0.016479

Functional classification of low expressed genes in co-cultured HPMVECs with MFLCs in comparison to PAFLCs

### Autophagic marker LC3 expression in human pulmonary microvascular ECs co-cultured with MFLCs

**Figure 6:**
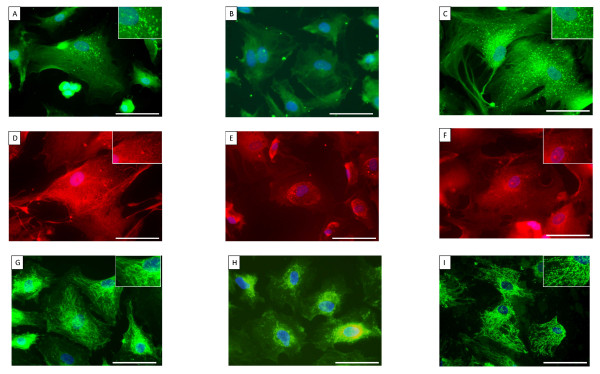
**Immunofluorescence staining of human pulmonary microvascular ECs (HPMVECs) and endothelial-like cells (ELCs) for the autophagic marker, LC3 (A-C), mitochondrial marker mitotracker red (D-F), and SOD-2 (G-I)**. A), HPMVECs co-cultured with PAFLCs; B), with MFLCs; C), with MFLCs + Rapamycin; A) and C), The formation of autophagosomes (green punctate structures) was detected (see inset). D), HPMVECs co-cultured with PAFLCs; E), MFLCs; F), with MFLCs + Rapamycin; D) and F), The normal filamentous mitochondrial reticulum (red punctate structures) was detected (see inset). G), HPMVECs co-cultured with PAFLCs; H), with MFLCs; I), with MFLCs + Rapamycin; G) and I), SOD-2 expression (green punctate structures) was detected (see inset). The blue staining was DAPI. The magnification was 400×. Scale bar = 25 μm. ELCs = endothelial-like cells.

Confocal microscopy showed that the ECs co-cultured with pulmonary arterial fibroblast-like cells expressed LC3. The formation of autophagosomes (green punctate structures) was detected in these cells (Figure 6A), but not in ECs co-cultured with MFLCs (Figure 6B) nor in ELCs (Figure 2K).

### Abnormal mitochondria and decreased expression of superoxide dismutase (SOD)-2 in human pulmonary microvascular ECs co-cultured with MFLCs

Immunofluorescence staining for mitochondrial marker mitotracker red revealed that the normal filamentous mitochondrial reticulum observed in ECs co-cultured with pulmonary arterial fibroblast-like cells (Figure 6D) was disrupted and rarefied in both ECs co-cultured with MFLCs (Figure 6E) and ELCs (Figure 2L). Moreover, SOD-2 was decreased in ECs co-cultured with MFLCs (Figure 6H) and ELCs (Figure 2M) compared to those co-cultured with pulmonary arterial fibroblast-like cells (Figure 6G). The decrease in SOD-2 expression in ECs co-cultured with MFLCs and ELCs might be associated with a reduction in SOD-2 activity.

### Endothelial cell biology PCR array of human pulmonary microvascular ECs co-cultured with MFLCs

These results, including the phenotypic alterations, inactivation of autophagy, and mitochondrial dysfunction, suggested that the endothelial cell biology is altered in patients with CTEPH. Therefore, an endothelial cell biology PCR array was done to further explore the effects of MFLCs on endothelial cell biology.

There were decreases in the expression of 15 and increases in the expression of 3 genes in ECs co-cultured with MFLCs in comparison to the expression in those co-cultured with pulmonary arterial fibroblast-like cells (Figure 5B). The 15 decreased genes were ANXA5, BCL2, CDH5, COL18A1, CX3CL1, ITGA5, ITGAV, ITGB1, MMP1, NPPB, PGF, PLA2G4C, PLAU, RHOB, and SOD1 (Table 2). CDH5, COL18A1, CX3CL1, ITGA5, ITGAV, ITGB1 and RHOB are related to endothelial cell activation as adhesion molecules. MMP1, NPPB, PLAU and RHOB are related to endothelial cell activation, and are part of the extracellular matrix (ECM) molecules. ANXA5 and PLAU are related to endothelial cell activation with regard to thrombin activity. PGF is related to angiogenesis. PLA2G4C and SOD-1 are both related to the endothelial cell response to stress.

**Table 2:** Endothelial Cell Biology PCR Array

Biological process description	Gene name	Gene symbol	Public ID	P-value
Endothelial Cell Activation: Thrombin Activity	Annexin A5	ANXA5	NM_001154	0.023464
Endothelial Cell Injury: Response to Stress Anti-Apoptosis	B-cell CLL/lymphoma 2	BCL2	NM_000633	0.000247
Endothelial Cell Activation: Adhesion Molecules	Cadherin 5, type 2 (vascular endothelium)	CDH5	NM_001795	0.003968
Endothelial Cell Activation: Adhesion Molecules	Collagen, type XVIII, alpha 1	COL18A1	NM_030582	0.004024
Endothelial Cell Activation: Adhesion Molecules	Chemokine (C-X3-C motif) ligand 1	CX3CL1	NM_002996	0.000779
Endothelial Cell Activation: Adhesion Molecules	Integrin, alpha 5 (fibronectin receptor, alpha polypeptide)	ITGA5	NM_002205	0.000147
Endothelial Cell Activation: Adhesion Molecules	Integrin, alpha V (vitronectin receptor, alpha polypeptide, antigen CD51)	ITGAV	NM_002210	0.02125
Endothelial Cell Activation: Adhesion Molecules	Integrin, beta 1 (fibronectin receptor, beta polypeptide, antigen CD29 includes MDF2, MSK12)	ITGB1	NM_002211	0.018399
Endothelial Cell Activation: Extracellular Matrix (ECM) Molecules	Matrix metallopeptidase 1 (interstitial collagenase)	MMP1	NM_002421	0.021783
Permissibility and Vessel Tone: Regulation of Blood Pressure Regulation of Vascular Permeability Angiogenesis: Negative Regulation of Angiogenesis Endothelial Cell Activation: Extracellular Matrix (ECM) Molecules	Natriuretic peptide precursor B	NPPB	NM_002521	0.041345
Angiogenesis: Other Genes Involved in Angiogenesis	Placental growth factor	PGF	NM_002632	0.029734
Endothelial Cell Injury: Response to Stress	Phospholipase A2, group IVC (cytosolic, calcium-independent)	PLA2G4C	NM_003706	0.014626
Endothelial Cell Activation: Extracellular Matrix (ECM) Molecules Thrombin Activity	Plasminogen activator, urokinase	PLAU	NM_002658	0.039877
Endothelial Cell Activation Adhesion Molecules Angiogenesis: Positive Regulation of Angiogenesis Endothelial Cell Activation: Adhesion Molecules Endothelial Cell Injury: Other Genes Related to Apoptosis	Ras homolog gene family, member B	RHOB	NM_004040	0.035874
Endothelial Cell Injury: Response to Stress	Superoxide dismutase 1, soluble	SOD1	NM_000454	0.001855

Functional classification of low expressed genes in co-cultured HPMVECs with MFLCs in comparison to PAFLCs

The 3 genes with increased expression were AGTR1, CASP1, and TIMP1 (Table 3). AGTR1 is related to the permissibility and vessel tone of the angiotensin system. CASP1 is related to endothelial cell injury and resulting apoptosis. TIMP1 is related to endothelial cell activation and cell growth.

**Table 3:** Endothelial Cell Biology PCR Array

Biological process description	Gene name	Gene symbol	Public ID	P-value
Permissibility and Vessel Tone: Angiotensin System	Angiotensin II receptor, type 1	AGTR1	NM_031850	0.030612
Endothelial Cell Injury: Caspase Activation	Caspase 1, apoptosis-related cysteine peptidase (interleukin 1, beta, convertase)	CASP1	NM_033292	0.005519
Endothelial Cell Activation: Other Genes Involved in Cell Growth	TIMP metallopeptidase inhibitor 1	TIMP1	NM_003254	0.049858

Functional classification of highly expressed genes in co-cultured HPMVECs with MFLCs in comparison to PAFLCs

### SMAD reporter signal in human pulmonary microvascular ECs co-cultured with MFLCs

The SMAD2 and SMAD3 proteins are phosphorylated and activated by TGF-β signaling. These activated SMAD 2 and SMAD 3 then form complexes with the SMAD4. These SMAD complexes then migrate to the nucleus, where they activate the expression of TGF-β-responsive genes.

Besides simple concentration measurements of TGF-β1 in the conditioned medium (Figure 3H), the activation of the TGF-β signaling in human pulmonary microvascular ECs co-cultured with MFLCs were measured by the SMAD reporter assay. There was no statistical difference in the expression of SMAD reporter signal in ECs co-cultured with MFLCs in comparison to the expression in those co-cultured with pulmonary arterial fibroblast-like cells (Figure 5C).

### Accumulation of ROS in human pulmonary microvascular ECs co-cultured with MFLCs

Accumulation of ROS coupled with an increase in oxidative stress has been implicated in the pathogenesis of numerous disease states. As SOD1 and SOD2 downregulation have been shown by the PCR-Arrays (Figure 5B) and immunofluorescence (Figure 6H), the missing production of ROS might be involved in ECs co-cultured with MFLCs [25]. The decreased production of ROS has been detected in ECs co-cultured with MFLCs in comparison to the expression in those co-cultured with pulmonary arterial fibroblast-like cells (Figure 5D).

### Rapamycin treatment

Prolonged rapamycin treatment of ECs co-cultured with MFLCs reversed the decrease in the 17 autophagy-related genes (Figure 5A) (Table 1) and prevented the changes in expression in 11 of the 15 decreased and all three of the increased genes related to endothelial cell biology (Figure 5B) (Table 2, 3). There was no statistical difference in the expression of SMAD reporter signal in ECs co-cultured with MFLCs with rapamycin (Figure 5C). Although rapamycin treatment of ECs co-cultured with MFLCs seemed to reverse the decreased production of ROS (Figure 5D), there was no statistical difference between them.

Confocal microscopy showed that the ECs co-cultured with MFLCs that were treated with rapamycin expressed LC3. Although the formation of autophagosomes (green punctate structures) was not detected in ECs co-cultured with MFLCs (Figure 6B), it was detected in these cells when they were treated with rapamycin (Figure 6C). In the ECs co-cultured with MFLCs, the co-localization of Mitotracker red and SOD-2 was lost, indicating that the mitochondrial reticulum is disrupted (Figure 6E, 2M). However, the mitochondria in the ECs co-cultured with MFLCs that were treated with rapamycin form an intricate, filamentous network, in which SOD-2 and Mitotracker red are tightly co-localized (Figure 6F, I).

## Discussion

EnMT is a term which has been used to describe the process through which ECs lose their endothelial characteristics and gain expression of mesenchymal, myofibroblast-like characteristics [26]. In the present study, a few transitional cells (co-expressing both endothelial- and SM- cell markers) were shown in the primary culture of endarterectomized tissue specimen (Figure 2C, F). The microenvironment created by the stabilized clot is suggested to induce EnMT (Figure 3F, G, 4D, E). Moreover, CD31 and α-SMA double-positive cells were identified in the neointimal layer of vascular wall tissue, thus indicating the presence of transitional cells in the neointimal lesions of CTEPH (Figure 2J). In support of our finding, Yao et al showed the presence of CD34 (endothelial marker) positive cells co-expressing α-SMA (SM-cell marker) in endarterectomized tissues from patients with CTEPH [27]. Moreover, they suggested that the microenvironment provided by thromboemboli might promote the putative progenitor cells to differentiate and enhance intimal remodeling [27]. In this study, our data suggest that MFLC-related EnMT may enhance intimal remodeling. However, we fully realize the limitations of our data interpretation, which was based on *in vitro *studies of cultured cells, and acknowledge that data provided in this study were not strong to support EnMT hypothesis because this study failed to show mechanisms responsible for this process. Moreover, it may be possible that transitional cells are more likely progenitor cells rather than they are transdifferentiated by EnMT.

There was no significant difference in TGF-β1 levels between serum-free medium conditioned by MFLCs and by pulmonary arterial fibroblast-like cells (Figure 3H). Moreover, there was no statistical difference in the expression of SMAD reporter signal in ECs co-cultured with MFLCs in comparison to the expression in those co-cultured with pulmonary arterial fibroblast-like cells (Figure 5C). A recent study provides evidence that Ras/MAPK, via TGF-β1 signaling, mediates completion of EnMT in a bleomycin model of pulmonary fibrosis [28]. However, an endothelial cell biology PCR array in this study demonstrated the decreased expression of RHOB (Ras homolog gene family, member B) in co-cultured human pulmonary microvascular ECs with MFLCs in comparison to pulmonary arterial fibroblast-like cells. These results suggest that not only TGF-β1 nor Ras, but also additional factors, may be essential for this transitional pathway. Indeed, TGF-β1 is currently thought to be insufficient to induce the late stage of SM differentiation in non-SMC lineage cells [24]. Moreover, neither TGF-β1 nor activated Ras alone were capable of inducing α-SMA expression [28].

The effects of conditioned media may be particularly remarkable if chemically defined culture media without serum additions is employed. Therefore, serum free media was adapted for the conditioned media experiments. However, this leads to serum starvation on the cells, which commonly leads to cell cycle arrest and induces changes in protein synthesis. Accordingly, co-culture experiments were conducted in media with serum, which allows different cell types to grow on either side of the membrane and may be able to detect the mutual effects of cell types on one another.

An inactivation of autophagy was found in both ELCs (Figure 2K) and human pulmonary microvascular ECs co-cultured with MFLCs (Figure 6B) compared to the expression in human pulmonary microvascular ECs co-cultured with pulmonary arterial fibroblast-like cells (Figure 6A), thus suggesting that in these cells, protein synthesis predominates over protein degradation. Moreover, the decreased expression of cell death-related genes indicated that cell growth may be stimulated (Figure 5A). This inactivation could benefit cancer cells. Recently several genetic links between autophagy defects and cancers have been shown, providing increasing support for the concept that autophagy is a genuine tumor suppressor pathway [29]. Signaling pathways that regulate autophagy overlaps with those that regulate tumorigenesis [16].

This study has shown that human pulmonary microvascular ECs co-cultured with MFLCs and ELCs have fewer mitochondria with an organized reticulum (Figure 6E, 2H) and SOD-2, which is an enzyme found only in the mitochondria, is decreased in these cells (Figure 6H, 2L). Endothelial cell biology PCR array demonstrated the decreased expression of SOD1 (Table 2), which is located in the cytoplasm. Both SOD1 and 2 are an important antioxidant defense in almost all cells exposed to oxygen. Moreover, the decreased production of ROS has been detected in ECs co-cultured with MFLCs in comparison to the expression in those co-cultured with pulmonary arterial fibroblast-like cells (Figure 5D). These results including fewer mitochondria, the decreased expression of SOD, and normoxic decreases in ROS are compatible with the characteristics of mitochondrial abnormalities in PAH, demonstrated by Archer et al [25]. The metabolic shift from oxidative mitochondrial metabolism to the glycolytic metabolism inhibits acetyl-CoA to enter the Krebs' Cycle, resulting in reduced production of ROS. However, gene and protein expression of SOD are not directly translated into activity and the decreased production of ROS is not sufficient to determine SOD activity. It has been shown that pulmonary artery SMCs in PAH are associated with mitochondrial disorders [30-32]. Xu and colleagues used an *in vitro *experiment with pulmonary artery ECs from idiopathic PAH (IPAH ECs) and control lungs (control ECs) to show that glucose metabolism plays the primary role in the energy requirements of IPAH ECs, based on the 3-fold greater glycolytic rate of IPAH ECs compared with control ECs. This indicates that there is mitochondrial dysfunction in ECs in patients with idiopathic PAH, similar to the SMCs in PAH [33]. The existence of mitochondrial disorder/dysfunction in commercially available pulmonary microvascular ECs co-cultured with MFLCs in CTEPH and ECs in PAH, may support the similarities in the microvascular remodeling in the two disease.

Although several protein kinases regulate autophagy, the mammalian target of rapamycin (mTOR), which negatively regulates the pathway in organisms from yeast to man, is the best characterized [15]. Rapamycin is an inhibitor of mTOR and an anti-proliferative immunosuppressor that arrests cells in the G1 phase of the cell cycle [34]. Rapamycin is used clinically in cardiovascular medicine as an anti-proliferative agent applied to coronary stents to reduce local restenosis [35]. Rapamycin inhibits hypoxia-induced activation of S6 kinase in pulmonary arterial adventitial fibroblasts [36], suggesting the possibility that there may be a therapeutic benefit in PAH. Moreover, rapamycin has an anti-proliferative effect on pulmonary arterial SMCs derived from endarterectomized tissues of CTEPH patients [37]. In this study, we demonstrated that rapamycin reversed the decrease in autophagy in the ECs co-cultured with MFLCs (Figure 5A, 6C). Moreover, rapamycin also reversed the disruption of the mitochondrial reticulum and restored the localization of SOD-2 (Figure 6F, I). It is acknowledged that mTOR activity antagonize induction of the general stress response genes including SOD-2 gene [38-40]. This may explain the mechanisms by which rapamycin exerts its beneficial changes on cellular mitochondria and SOD2 expression. Indeed, SOD2 is located within the mitochondrial matrix and was strongly induced in response to rapamycin in normal and neoplastic mammalian cells [41]. It also reversed the change in expression of 11 of the 15 genes decreased by co-culture and the 3 genes increased by co-culture that were related to endothelial cell biology (Figure 5B) (Table 2, 3), thus suggesting that rapamycin (as an anti-proliferative agent) has beneficial therapeutic effects, not only on pulmonary arterial SMCs, but also on pulmonary arterial ECs which exist in the close contact with MFLCs, in the patients with CTEPH. However, because rapamycin may act on the proliferation rate of MFLCs more than pulmonary arterial fibroblast-like cells [37], it is possible that this action may be an alternative explanation for the observed differences. Moreover, a few transitional cells were observed in the ECs co-culture with MFLCs that were treated with rapamycin (data not shown), indicating that rapamycin might exert no beneficial effect on EnMT.

## Conclusions

**Figure 7:**
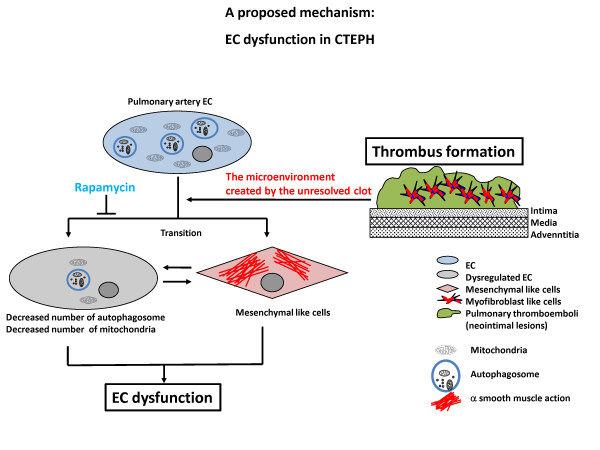
**EC dysfunction in CTEPH (a proposed mechanism)**. Our experiments with ECs and MFLCs demonstrated that the microenvironment provided by the thrombus cells in CTEPH patients might induce EC dysfunction through EnMT, inactivation of autophagy, disruption of mitochondrial reticulum, and the improper SOD-2 localization, although it remains unknown whether both EnMT and other cell function alterations are taking place simultaneously in the same ECs. Although it is uncertain whether MFLCs induce EC dysfunction *in vivo *and whether EC dysfunction contribute to the vascular lesions in the patients with CTEPH, it is possible that there exist dysfunctional ECs in the microenvironment created by the unresolved clot.

Our experiments with ECs and MFLCs demonstrated that factors associated with MFLCs in the microenvironment created by the unresolved clot might induce EC dysfunction through EnMT (3F, 3G, 4D, 4E), inactivation of autophagy (Figure 5A, 6B), disruption of the mitochondrial reticulum, and improper localization of SOD-2 (Figure 6E, H). Indeed, ELCs, which were isolated from the PEA tissues of CTEPH patients, included a few transitional cells (coexpressing both endothelial- and SM- cell markers) (Figure 2J), lost their ability to form autophagosomes (Figure 2K) and had defective mitochondrial structure/function (Figure 2L). Although it is uncertain whether MFLCs induce EC dysfunction *in vivo *and whether EC dysfunction contribute to the vascular lesions in the patients with CTEPH, it is possible that there exist dysfunctional ECs in the microenvironment created by the unresolved clot (Figure 7). However, non-resolving pulmonary thromboemboli in CTEPH mainly consist of fibrotic tissue representing the end-stage of a thrombus organization process. Therefore, it remains uncertain whether any of the cellular or molecular findings at this stage of disease are causally involved in disease pathogenesis. We should acknowledge the purely descriptive nature of this study that does not confer any pathophysiological evidence in CTEPH.

## Competing interests

Dr. Tatsumi has received honoraria for lectures from Glaxo Smith Kline, Actelion Pharmaceutical Ltd. Dr. Tanabe has received honoraria for lectures from Actelion, Glaxo Smith Kline, Astellas and Pfizer and research grant support from Actelion Pharmaceutical Ltd. The other authors report no conflicts.

## Authors' contributions

SS conceived of the report, contributed to its design and conception, drafted the manuscript and carried out the all studies. HH carried out the pathological studies. NT drafted the manuscript and contributed to its design and conception. YK contributed to its design. KK carried out the pathological studies. KT contributed to its design and drafted the manuscript. All authors read and approved the final manuscript.
